# Spatiotemporal dynamics of *Plasmodium falciparum* histidine-rich protein 2 and 3 deletions in Peru

**DOI:** 10.1038/s41598-022-23881-8

**Published:** 2022-11-18

**Authors:** Hugo O. Valdivia, Karen Anderson, David Smith, Cielo Pasay, Carola J. Salas, Greys Braga, Carmen M. Lucas, Stephen E. Lizewski, Christie A. Joya, Jennifer M. Kooken, Juan F. Sanchez, Qin Cheng

**Affiliations:** 1 U.S. Naval Medical Research Unit No. 6 (NAMRU-6), Lima, Peru; 2 Australia Defence Force Malaria and Infectious Disease Institute, Brisbane, Australia; 3 QIMR-Berghofer Medical Research Institute, Brisbane, Australia; 4 Walter Reed Army Institute for Research, Silver Spring, USA

**Keywords:** Malaria, Population genetics

## Abstract

Peru was the first country where *pfhrp2* and *pfhrp3* gene deletions were detected despite the fact that rapid diagnostics tests are not commonly used for confirmatory malaria diagnosis. This context provides a unique scenario to study the dynamics of *pfhrp2* and *pfhrp3* gene deletions without apparent RDTs selection pressure. In this study we characterized the presence of *pfhrp2* and *pfhrp3* genes on 325 *P. falciparum* samples collected in Iquitos and surrounding communities between 2011 and 2018 in order to understand the dynamics of gene deletion prevalence, potential associations with clinical symptomatology and parasite genetic background. *P. falciparum* presence was confirmed by microscopy and PCR of 18 s rRNA, *pfmsp1* and *pfmsp2*. Gene deletions were assessed by amplification of exon1 and exon2 of *pfhrp2* and *pfhrp3* using gene specific PCRs. Confirmation of absence of HRP2 expression was assessed by ELISA of HRP2 and pLDH. Genotyping of 254 samples were performed using a panel of seven neutral microsatellite markers. Overall, *pfhrp2* and *pfhrp3* dual gene deletions were detected in 67% (217/324) parasite samples. Concordance between *pfhrp2* deletion and negligible HRP2 protein levels was observed (Cohen's Kappa = 0.842). Prevalence of gene deletions was heterogeneous across study sites (adjusted p < 0.005) but there is an overall tendency towards increase through time in the prevalence of dual *pfhrp2/3*-deleted parasites between 2011 (14.3%) and 2016 (88.39%) stabilizing around 65% in 2018. Dual deletions increase was associated with dominance of a single new parasite haplotype (H8) which rapidly spread to all study sites during the 8 study years. Interestingly, participants infected with dual *pfhrp2/3*-deleted parasites had a significantly lower parasitemias than those without gene deletions in this cohort. Our study showed the increase of *pfhrp2/3* deletions in the absence of RDTs pressure and a clonal replacement of circulating lines in the Peruvian Amazon basin. These results suggest that other factors linked to the *pfhrp2/3* deletion provide a selective advantage over non-deleted strains and highlight the need for additional studies and continuing surveillance.

## Introduction

Malaria is an infectious disease caused by parasites of the *Plasmodium* genus that is widely distributed across 87 countries in tropical and subtropical regions^1^. Despite progress towards malaria control, recent estimates indicate that malaria has caused 241 million cases and 627,000 deaths in 2020^2^. In the Americas, progress towards malaria elimination has resulted in a case reduction of nearly 40% from 1.5 million cases in 2000 to 0.9 million in 2019. However, more than 86% of all cases in 2019 were reported in Venezuela, Colombia and Brazil^1^.

Current malaria guidelines established by WHO in 2010 recommend that all suspected malaria cases should have a microscopy or rapid diagnostic test to confirm the diagnosis before treatment with antimalarial drugs^3^. Consequently, antigen-based rapid diagnostic tests are key for accomplishing this recommendation and to expand access to point of care malaria diagnosis in endemic areas where microscopy cannot be performed^3^.

Most RDTs available target (i) the histidine-rich protein 2 (HRP2) for *P. falciparum* specific detection and (ii) lactate dehydrogenase or aldolase for species specific or pan-specific detection of the more relevant *Plasmodium* species (*P. falciparum*, *P. vivax*, *P. malariae* and *P. ovale*)^4^. However, their sensitivity and utility is challenged by the emergence and spread of *pfhrp2* and *pfhrp3* deleted parasites.

The first evidence of deletions on *pfhrp2* and *pfhrp3* came from Peru in 2010 when 148 *P. falciparum* samples were tested by PCR and ELISA^5^. Results of that study showed that out of 148 samples, 61 were *pfhrp2* deleted (41%), 103 *pfhrp3* deleted (70%) and 32 were dual *pfhrp2/3* deleted (21.6%). Subsequent studies have shown that *pfhrp2/3* deletions are not only prevalent in South America^6,7^ but are also present in endemic regions in Africa, Asia and middle east^8–13^.

Since 2009, WHO has published the results of malaria RDT product testing for a systematic comparison of the performance of commercially available RDTs^4^. In this regard, results of the latest evaluation from 2018 showed that HRP2 and HRP2-pan-LDH RDTs can lead to misdiagnoses or misclassification of malaria infections in areas with circulating *pfhrp2/3-*deleted *P. falciparum* parasites^4^. The increasing evidence of the extend of *pfhrp2/3* deletions and their implications for accuracy of malaria diagnosis poses a major threat to malaria control and case management and underscores the need for continuing surveillance of *pfhrp2/3* gene deletions in endemic regions^4^.

Although RDTs are widely used in several countries in Africa and Asia, their use is limited in Peru which favors microscopy based diagnosis at the core of its malaria control strategy as directed by the country’s case management policy. Unfortunately, despite being the source of the first report of *pfhrp2/3* deletions, recent data is lacking regarding status of these deletions in Peru to reveal changes in prevalence and evolution of parasites since the original reports.

While RDT use may exert a strong selection pressure on local parasite population by selecting for RDT-undetectable parasite strains, drug resistance and treatment policy changes can certainly drive parasite population changes. During the 1990’s, Peru experienced a major public health crisis due to malaria, reaching a peak of 120,000 microscopy confirmed cases in 1997^14^. Subsequent studies showed that *P. falciparum* parasites from the coast and western Amazon were resistant to chloroquine (CQ) but sensitive to sulfadoxine pyrimethamine (SP), whereas strains from the rest of the Amazon were CQ and SP resistant^15,16^. In response to these studies, Peru implemented in 2001 artesunate plus mefloquine combination therapy (ACT) in the Amazon region and ART plus SP for the coastal region^15^. This change and increased control activities reduced the annual incidence of malaria in the Amazon (Loreto region) at around 45,000–55,000 cases between 2002 and 2005. During that time, Peru also enhanced several control strategies within the Global Fund PAMAFRO project^17,18^. This project was executed between 2005 and 2010^19^ and led to a drastic reduction on the number of malaria cases to 23,000 cases in 2011^18,20,21^. The end of PAMARO in 2010 put a hold to the progress achieved towards malaria elimination which was reflected on an increase in the number of cases up to 55,000 cases in 2017^22^.

The rapid reduction of cases due to treatment policy change and intensified control activities had an impact on circulating parasite genetics. In this regard, a study conducted with samples from 1999 and 2000 showed the presence of five *P. falciparum* strains (A, B, C, D, E) that circulated in the Amazon region of Loreto^23^. A subsequent analysis with samples from 2006 and 2007 showed a reduction in the prevalence of previous strains in favor of new hybrid derived from strains B and C or C and D (B/C and C/D)^23^.

In addition, a subsequent *P. falciparum* outbreak in the North Peruvian coast showed that a single strain that was introduced from Loreto was responsible for the outbreak^24^. This strain was related to the B strain reported in Loreto but was not completely identical and therefore was referred as Bv1. This strain presented several drug resistance associated mutations in *pfdhfr, pfdhps, pfcrt y pfmdr1* and seemed to have deleted the *pfhrp2* gene^24^.

This highly dynamic scenario highlight the need of time trend data that could (i) provide hints into changes in *P. falciparum* epidemiology, (ii) changes in circulating parasite populations and (iii) temporal dynamics and evolution of *pfhrp2/3* deleted and non-deleted parasites in an area where there is an apparent absence of RDT derived selection pressures but had undertaken intensified malaria control interventions.

## Methods

### Study design

**Figure 1:**
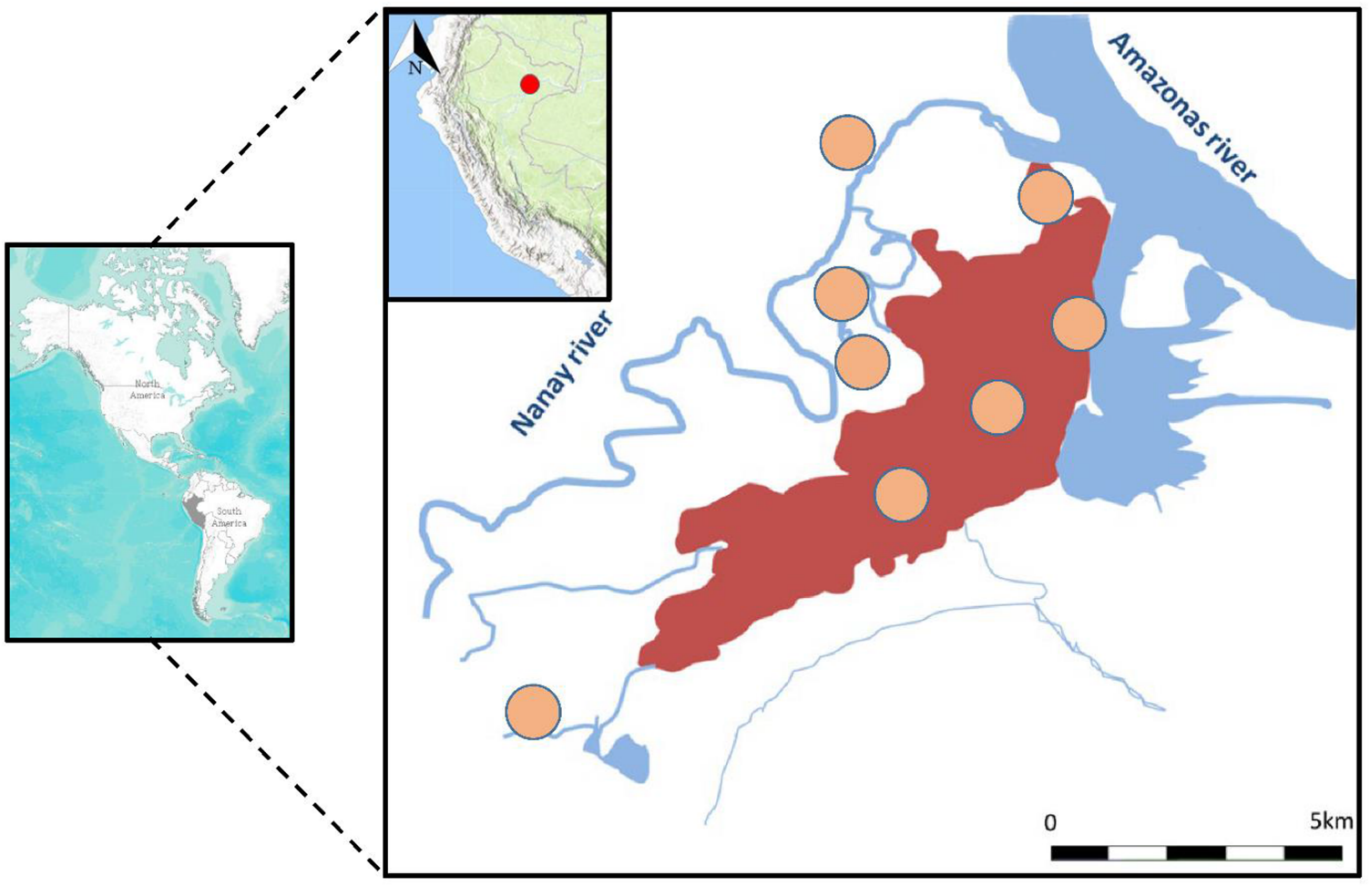
Collection sites. Study sites located in the city of Iquitos and surrounding communities. Orange circles showed the different health centers where samples were collected. The map was created using open data obtained from GADM database of Global Administrative Areas, version 3.6. URL: https://www.gadm.org.

Samples for this study were collected as part of a passive surveillance protocol conducted in eight study sites (Amazon Hope, Bellavista Nanay, Apoyo Hospital, Moronacocha,

Padrecocha, Puerto America, Santa Clara and Tupac) in the city of Iquitos and surrounding communities located in the region of Loreto in the Northern Amazon Basin (Fig. 1). This region is a tropical area with an annual average temperature of 27 °C, 80% humidity and 4 m annual rainfall. According to Peru’s Ministry of Health, Loreto reported 15,235 malaria cases in 2020 of which 3,049 (20%) were *P. falciparum* and 12,186 *P. vivax* (80%)*.*

### Human ethics considerations

The protocol for this study was approved by the Institutional Review Board of the U.S Naval Medical Research Unit 6 (NAMRU-6) in compliance with all applicable federal regulations governing the protection of human subjects (protocol NMRCD.2007.0004). Informed consent was obtained from all participants. For participants aged 8 to 17 years, written consent from parents or guardians and assent from participants were obtained.. The laboratory analysis of blood samples at the Australia Defence Force Malaria and Infectious Disease Institute (ADFMIDI) was approved by the Department of Defence and Veterans’ Affairs Human Research Ethics Committee (DDVA HREC 213-20).

### Study population

The study enrolled individuals referred to one of the study sites with suspected or confirmed malaria. Inclusion criteria were limited to participants older than one year old with documented fever or history of fever during the previous 72 h in the absence of another cause of fever such as pneumonia or acute otitis media and who signed informed consent and assent. As part of enrolment procedures, clinical and epidemiological data were collected from each participant on a case report form.

### Study procedures

**Figure 2:**
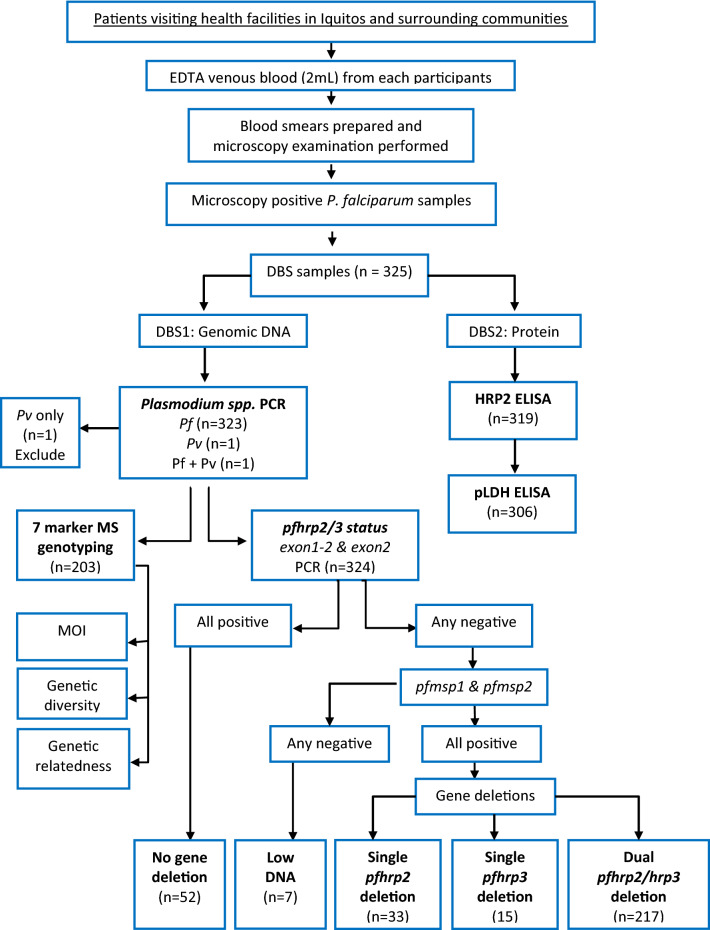
Flow chart illustrating study procedures.

Two mL of EDTA-whole blood were collected by venipuncture from each participant. Blood was used to prepare two thin and thick smears, stained with 10% Giemsa and read by two study microscopists in the NAMRU-6 laboratory in Iquitos. Parasite densities were determined by counting the number of parasites per 500 white blood cells on blood films and converted to parasites/µL blood based on a white blood cell count of 8000 cells/µL. Remaining whole blood was aliquoted and stored at −80 ºC for further testing. Dried blood spots (DBS) from microscopy positive *P. falciparum* samples (n = 325) collected between 2011 and 2018 were prepared on Whatman Protein Saver Cards (two spots of 50µL blood per participant) and sent to ADFMIDI for subsequent molecular and serological detection of *pfhrp2/3* gene deletions, microsatellite and genotyping. The study procedures are illustrated in a flow chart in Fig. 2.

### DNA extraction

Genomic DNA was extracted from one DBS using the QIAamp^®^ DNA Mini Kit and a QIAcube Connect (GmbH, Hilden, Germany) following the manufacturer’s instructions. Genomic DNA was eluted into 100 µL and stored at −20 °C until use.

### Confirmation of Plasmodium infection

Plasmodium infection was confirmed for each sample using 5 µL of genomic DNA in a multiplex PCR targeting parasite 18S rRNA gene^25^.

### Determination of *pfhrp2* and *pfhrp3* gene status

*pfhrp2* and *pfhrp3* gene deletions were determined by conventional PCRs that amplifies exon1 and exon2 of *pfhrp2* and *pfhrp3* in four separate reactions. Each PCR was carried out in a single round of 45 cycles using conditions described previously^5^. Amplification of *pfmsp1* and *pfmsp2* was also carried out as a quality control for DNA in compliant with recommendations^26^. Samples were classified as having *pfhrp2* and *pfhrp3* gene deletions only if they failed to amplify exon1 and/or exon2 of these genes but amplified both *pfmsp1* and *pfmsp2* genes.

### Expression of HRP2 and pLDH

The second DBS was used to elute proteins using a previously described method^27,28^ with a modification of eluting at room temperature for 3 h, instead of at 12 °C for 12 h. Proteins were eluted into 200 µL and stored at −20 °C until use. Eluate (100 µL each) was used to measure the expression of HRP2 (Quantimal Celisa Pf HRP2 Assay kit, Cellabs, Cat number: KM 810) and pLDH (Quantimal Celisa Pf pLDH Assay kit, Cellabs, Cat number: KM7) following manufacturer’s instructions. Uninfected DBS were prepared in the laboratory by spotting 50µL blood collected from healthy donors by the Australian Red Cross Blood Services Brisbane, and air dried. Proteins were also eluted from uninfected DBS and used in each ELISA as negative controls. ΔOD450nm (OD450nm sample—OD450nm uninfected human blood) was used to represent the quantity of HRP2 and pLDH in the DBS sample.

### Microsatellite typing

Genotyping was carried out for a subset of samples (n = 254) by amplifying seven neutral microsatellite markers (TA1, PolyA, PfPK2, TA109, 2490, 313, and 383) and by determining length of each amplified marker. A semi-nested PCR was used to amplify five of the seven microsatellite markers (TA1, PolyA, PfPK2, TA109 and 2490), while a single round RCR was used to amplify the remaining two of the seven markers (313 and 383) using published primers and PCR conditions^29,30^ . Fluorescent labelled PCR products were analysed on an ABI 3100 Genetic Analyzer sequencer (Applied Biosystems) to determine their length. Peak Scanner Software version 1.0 (Applied Biosystems, https://peak-scanner-software.software.informer.com/1.0/) was used to manually score peaks. A peak height > 300 relative fluorescence units (rfu) was considered as a positive peak^11^. For samples producing more than one peak, the highest peak was defined as the dominant allele in the sample while minor peaks were defined as minor alleles if their peak heights were > 300 rfu and > 30% of the highest peak.

### Haplotype frequency

Seven-microsatellite-marker-haplotypes were constructed for samples with positive peaks at all seven markers. Unique haplotypes were identified and assigned a unique number. The number of samples belonging to each unique haplotype is used to calculate haplotype frequency.

### Data analysis

ELISA data was analysed using GraphPad Prism (version 9) and the package cutpointr implemented in R for calculation of the optimal cutpoint, sensitivity and specificity^31^.

Microsatellite data was used to assess polyclonal samples and multiplicity of infections. Data from monoclonal samples was used for discriminant analysis of principal components with and without k-means clustering using the adegenet package implemented in R^32^ as well as for genetic relatedness analysis on PHYLOViZ v1.1 using a cutoff of 2 (≥ 5/7 markers identical)^33^.

Clinical and epidemiological was analyzed in Stata 16. Human clinical data for deleted and non-deleted parasite populations was analyzed using Fisher's exact test or Kruskal–Wallis test to assess potential associations with disease severity, socio-demographic data or laboratory results.

## Results

### Study population

A total of 325 samples from participants from 2011 until 2018 were collected from eight health centers in Iquitos and surrounding communities. One sample was excluded from further testing as the PCR did not show infection with *P. falciparum*.

Complete demographic and clinical data was obtained from 317 participants. The missing data from 8 participants was due to missing information in their case report forms. The median participant age was 36 years old (IQR: – 2.6 to 81) and 64% (203/317) were males. Previous malaria episodes were reported on 69% (210/304) of participants with an average of 3 episodes in the last 10 years. Most participants 37.6% (111/295) reported to have a malaria case in their family in the previous year (Table 1).

**Table 1:** Sociodemographic and household characteristics of enrolled participants.

Characteristics	Falciparum malaria (n = 317)
**Socio-demographic data**	
Age (in years)^a^	36 (2.6–81)
Male (%)	203 (64%)
**Occupation**	
Agriculture	77 (24.3%)
Housewife	66 (20.8%)
Student	33 (10.4%)
Independent	24 (7.6%)
Other	117 (36.9%)
**Health facility (H.F.)**	
Hospital de Apoyo	117
Bellavista-Nanay HF	10
Moronacocha HF	24
Padrecocha HF	137
Tupac HF	2
Santa Clara HF	12
America HF	5
Amazon Hope	10
**Clinical data**	
Headache	298 (94%)
Malaise	281 (88.6%)
Chills	263 (92.9%)
Jaundice	174 (54.9%)
Fever	296 (93.6%)
Sweating	227 (74.4%)
Diarrhea	30 (9.8%)
Vomiting	20 (6.3%)
Unconcious	3 (0.9%)
Seizures	8 (2.5%)
Breathing problems	45 (14.2%)
**Laboratory data**	
Asexual parasitaemia (par/μL)^a^	3,991 (0–76,559)
Sexual parasitaemia (par/μL)^a^	0 (0–2319)
**Epidemiologic data**	
Malaria episodes at last 10 years^a^	2 (0–20)
Malaria episodes at last year^a^	1 (0–9)
Family member with malaria at last year	111 (37.6%)

^a^Median (IQR).

### Prevalence of *pfhrp2*/3 deletions

**Figure 3:**
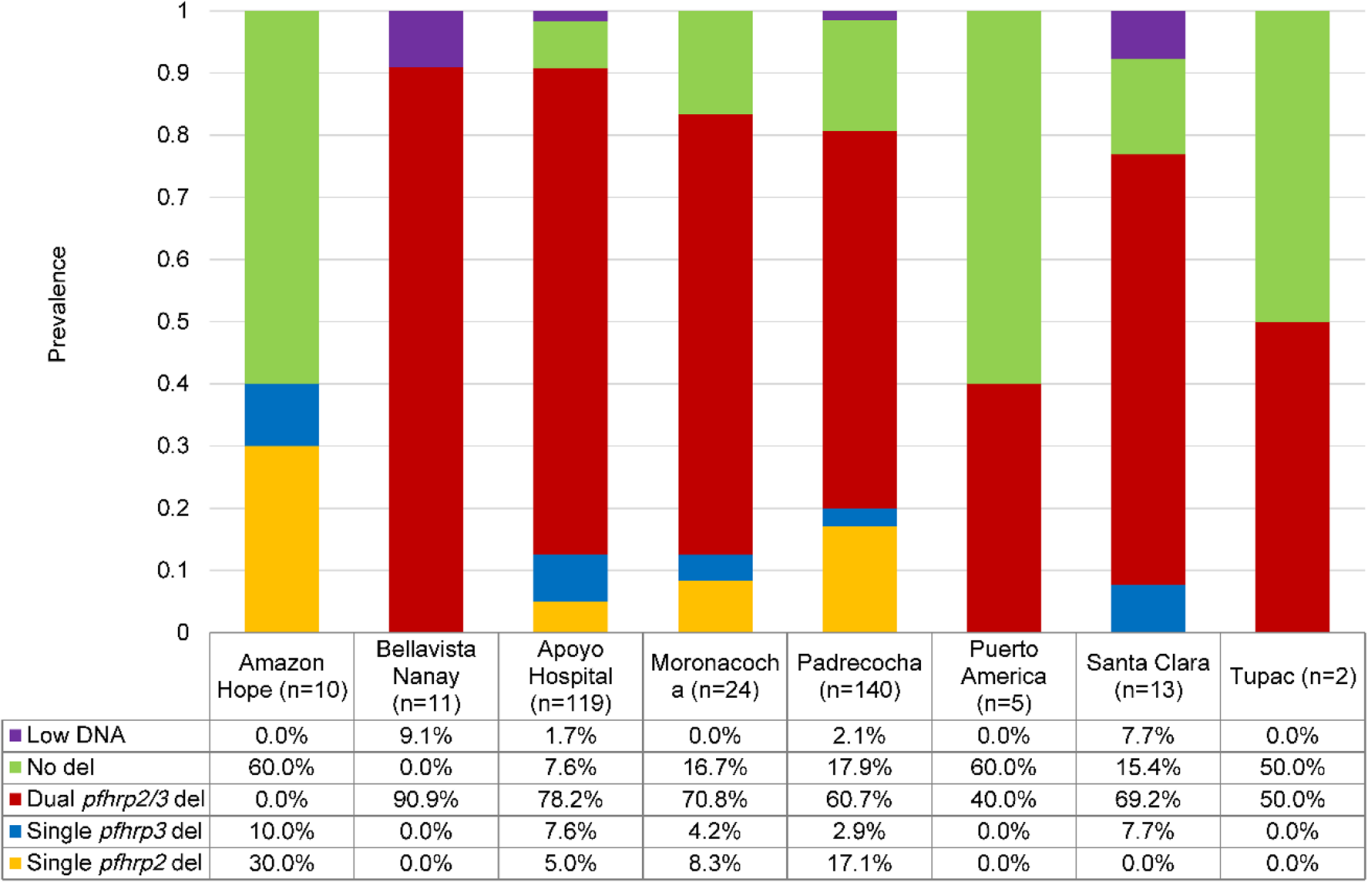
Prevalence of *pfhrp2* and *pfhrp3* deletions in Iquitos and surrounding areas. The figure shows the prevalence of single, dual and non-deleted parasites for *pfhrp2* and *pfhrp3*. Significant differences were found in the prevalence of Amazon Hope compared to Bellavista, Apoyo Hospital, Moranococha, Padrecocha and Santa Clara and for Apoyo Hospital against Padreocha according to pairwise Fisher’s exact test. Tupac and Puerto America were excluded from statistical analysis due to small sample numbers.

Infection with *pfhrp2* and/or *pfhrp3* negative parasites was common among the 324 samples that underwent PCR screening with 250 (77.2%) lacking *pfhrp2*, 232 (71.6%) lacking *pfhrp3* and 217 (67.0%) lacking both *pfhrp2* and *pfhrp3* (S1 Table). Significant lower gene deletion prevalence were found from samples collected in Amazon Hope compared to Bellavista, Apoyo Hospital, Moranococha, Padrecocha and Santa Clara (adjusted p < 0.005), and for Padrecocha against Apoyo Hospital (adjusted p < 0.005) based on false discovery rate corrected pairwise Fisher’s exact tests (Fig. 3). Patterns of gene deletions were observed with 81.6% *pfhrp2* deletions and 87.1% *pfhrp3* deletions presenting both exons deleted (S1 Table). Proportions of single exon1 and single exon2 deletions in *pfhrp2* and *pfhrp3* are also summarized in the S1 Table.

### Parasitemia and gene deletions

Analysis of *pfhrp2/3* deletion status and clinical/laboratory variables found a significant association between microscopy determined parasitemia levels and the presence of deletions (Kruskal–Wallis chi-squared p < 0.05) with dual *pfhrp2/3*-deleted samples presenting significantly lower mean parasitemia levels than non-deleted samples based on Pairwise Wilcoxon rank sum test with Benjamini–Hochberg FDR correction (p = 0.032, Fig. S1).

### ELISA confirmation of *pfhrp2/3* deletions

**Figure 4:**
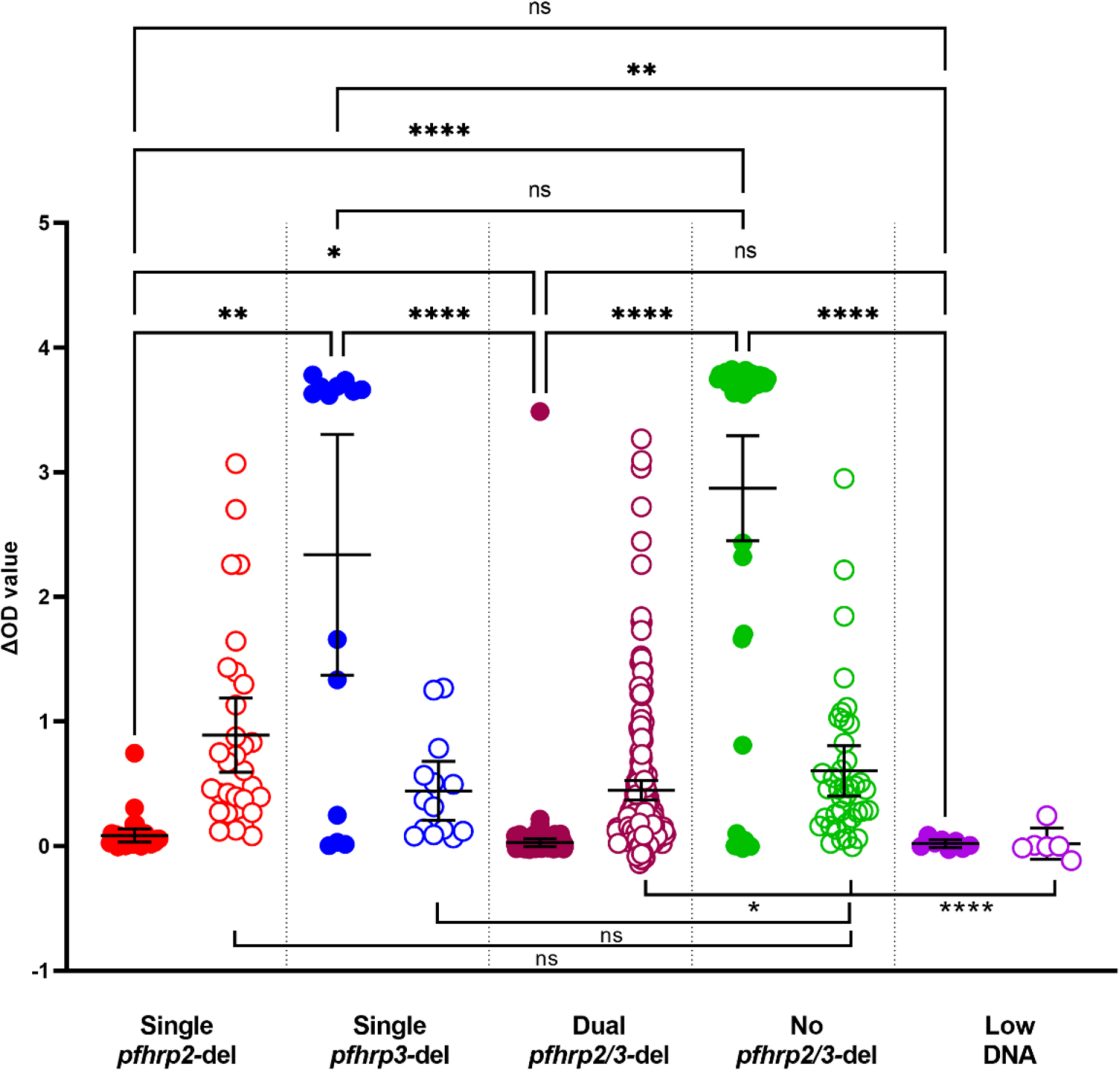
Comparison of HRP2 and pLDH protein levels in parasites with different *pfhrp2* and *pfhrp3* status. Solid dots: HRP2; circles: pLDH.

Parasite samples determined to have deleted a single *pfhrp2* gene or dual *pfhrp2/3* genes have negligible HRP2 protein except one sample in each group. Parasite samples determined to have deleted a single *pfhrp3* gene produced comparable levels of HRP2 compared to samples with no gene deletions (Fig. 4). Dual *pfhrp2/3-*deleted parasites had a significantly lower level of pLDH compared to parasites without gene deletions (Mann–Whitney test, p = 0.0116), consistent with microscopy results showing a lower parasitemia in dual *pfhrp2/3-*deleted sample set. Single *pfhrp2-* and *pfhrp3-*deleted parasites produced comparable levels of pLDH to parasites without gene deletions (Mann–Whitney tests, p > 0.05) (Fig. 4). Samples determined to have low DNA (low parasitemia) had negligible levels of both HRP2 and pLDH.

The optimal ELISA cutoff using *pfhrp2* PCR as reference test for the included data set was 0.5487 resulting in a sensitivity of 79.69% and specificity of 99.19% HRP2 detection. Using this cutoff point, significant agreement was found between HRP2 PCR and ELISA results (Cohen's Kappa = 0.842).

### Temporal distribution of *pfhrp2/3* deletions

**Figure 5:**
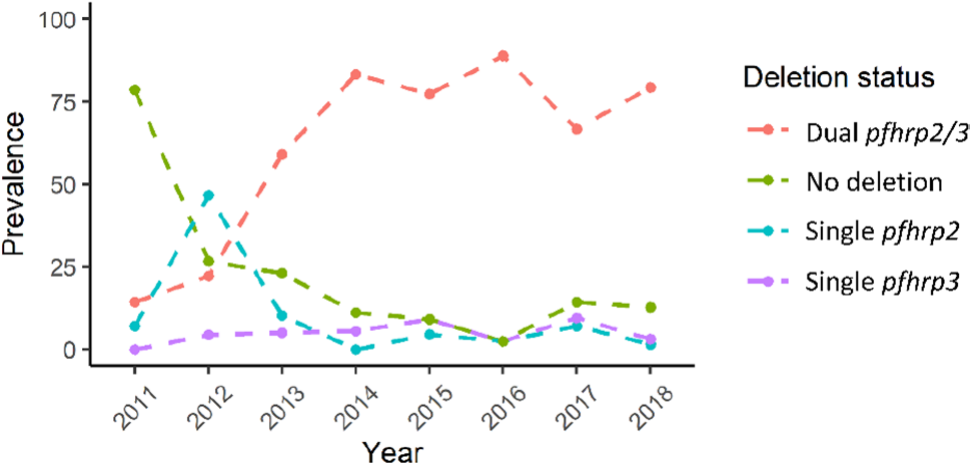
Temporal distribution of *pfhrp2* and *pfhrp3* deletions between 2011 and 2018. The figure shows a continuous increase the prevalence of the double *pfhrp2/3* deletion across time.

Statistical significant differences were found in dual *pfhrp2/3* deletion prevalence between 2011 and 2012 versus subsequent years (Fisher FDR adjusted p = 0.001) and between 2016 and 2017 (Fisher FDR adjusted p = 0.013). In this regard, the prevalence of single *pfhrp2*-deleted parasites in this sample set started at 7.1% in 2011, drastically increased to 46.7% in 2012 (S2 Table, Fig. 5) and subsequently decreased to 10.3% in 2013 down to less than 2% in 2018. The prevalence of single *pfhrp3*-deleted parasites remained under 10% across all years. In contrast, our results point towards an increase through time in the prevalence of the dual *pfhrp2/3*-deleted parasites starting at 14.3% in 2011 up to a peak in 2016 at 88.39%. The prevalence of dual deletions slowly decreased but remained over 65% in 2017 and 2018. The prevalence trend analyzed for two study sites where a relatively large number of samples was collected over the survey years, Apoyo Hospital (n = 119) and Padrecocha (n = 140), was similar to the overall trend (Fig. S2).

### Microsatellite genotyping and genetic diversity

Seven microsatellite markers were successfully typed on 203 out of 254 samples tested. Out of those, only 5 samples from the community of Padrecocha were polyclonal (2.5%) with 4/5 presenting a mixture of *pfhrp2−/pfhrp3*+ parasites whereas the remaining sample had a mixture of dual *pfhrp2/3* deleted parasites. The overall multiplicity of infection in this sample set was 1.025.

### Haplotypes dynamics

**Figure 6:**
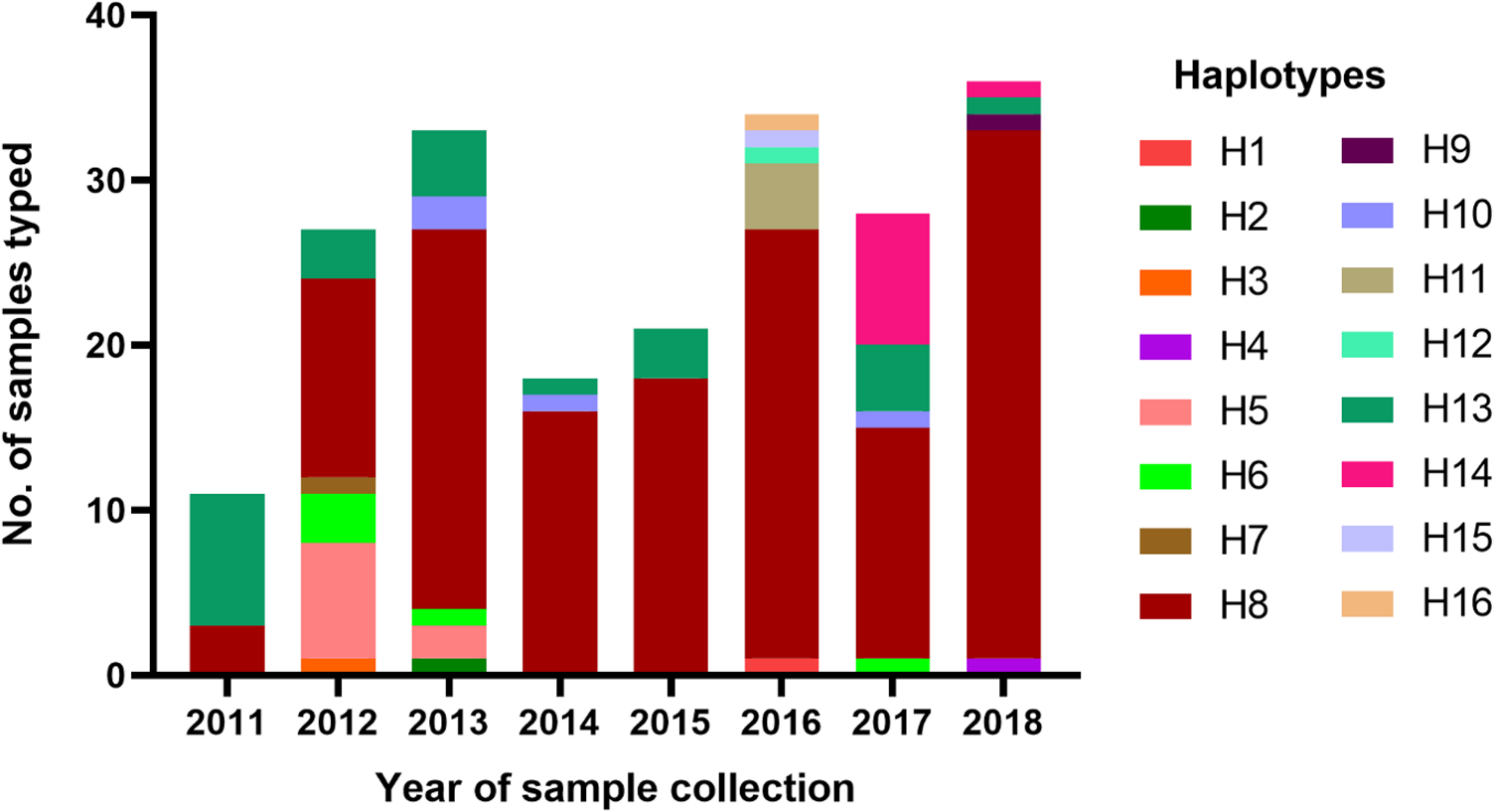
Dynamics of parasite haplotypes between 2011 and 2018.

A total of 208 haplotypes (203 single clone + 5 polyclonal) were constructed from 203 samples, of which 16 unique haplotypes were obtained. The most dominant haplotype, H8, was shared by 144/203 (70.9%) samples genotyped; while the second dominant haplotype, H13, was shared by 24/203 (11.8%) samples typed (Fig. S3). These two dominant haplotypes were the only haplotypes detected in samples collected in 2011 and both were detected in samples collected in all subsequent years except H13 in 2016. Over the 8 years, H8 became the predominant haplotype circulating in the study area, reaching 88.9% in 2018 (Fig. 6). This haplotype was initially detected in Padrecocha in 2011, then, in Padrecocha and Apoyo between 2012 and 2017. In 2018, it was detected in all five study sites (Padrecocha, Moronacocha, Santa Clara, Bellavista Nanay and Tupac) where samples were collected during this year. Importantly, of the 144 samples sharing H8, 120 (83.3%) were dual *pfhrp2/pfhrp3*-deleted parasites, followed by 14 (9.7%), 3 (2.1%) and 7(4.9%) of single *pfhrp2*-deleted, single *pfhrp3*-deleted and wild type parasites, respectively. In contrast, the second dominant haplotype H13, which was shared by 24 samples having intact *pfhrp2/3* genes (wild type), has shown reduced prevalence although present in most years. Therefore, the clonal expansion of H8 haplotype over time provides an explanation on the rise of prevalence of dual *pfhrp2/pfhrp3*-deleted parasites in the study area.

### Population structure and genetic relatedness

**Figure 7:**
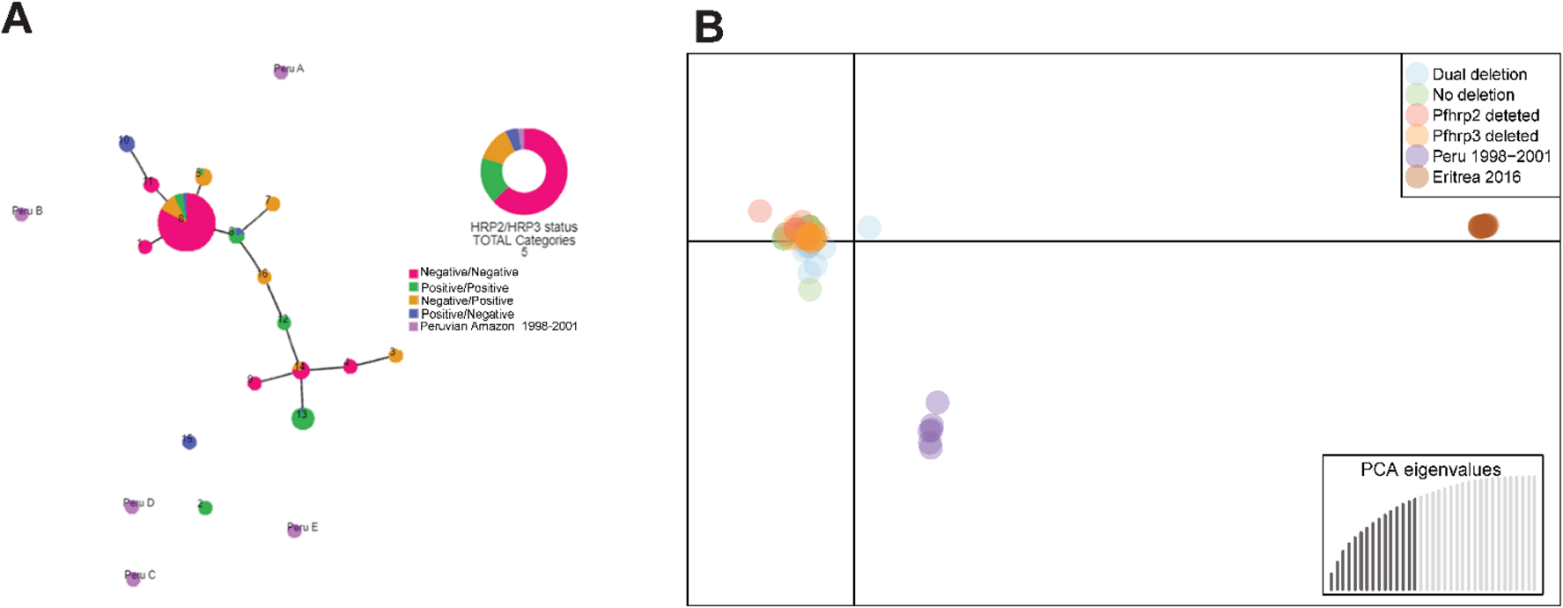
Genetic relatedness of parasites with different *phrp2/3* status. (**A**) PHYLOViZ showing that that most dual deleted parasites grouped in a single cluster and (**B**) DAPC result showing that samples from this study collected between 2011 and 2018 do not cluster with Peruvian samples collected in the early 2000s. Brown circles are samples from a published study from Eritrea which were used as controls for the analysis^11^.

Discriminant analysis of principal components, clustering and genetic relatedness analysis on PHYLOViZ showed that *pfhrp2*/3-deleted parasites from this study formed one cluster, but did not cluster with those collected in earlier years in Peru, indicating a new lineage of parasite circulating in the study area (Fig. 7A,B). Genetic relatedness analysis did not show differences in clustering according to temporal nor geographical location (Fig. S4A,B). In addition, DAPC with k-means clustering and PHYLOViZ showed that 91.6% (120/131) of dual *pfhrp2/3*-deleted parasites shared the same haplotype (H8) and furthermore, 100% of dual *pfhrp2/3*-deleted parasites are closely connected (Fig. 7A, Fig. S5). Clustering analysis showed that there was a significant difference in the proportions of deleted and non-deleted parasites belonging to each cluster with 90% of dual or single deleted parasites grouping in cluster 1 and 67.5% of non-deleted parasites belonging to cluster 2 (p < 0.001). In addition, parasites of different *pfhrp2/3* status shared same haplotypes or were closely connected in the one cluster suggesting they shared the same genetic background. These results further support that these parasites likely evolved from a single genetic background as a clonal expansion and that this parasite strain has persisted over time.

## Discussion

This is the first study on the trend and evolution of *pfhrp2/3* deleted parasites at a single location over a decade. Although Peru was the first country where *pfhrp2/3* deleted parasites were reported^5^, temporal data was lacking. Therefore, this study collected samples over the years from the same study location in order to provide comparable data.

In contrast to what is seen in African countries, RDTs are not the main diagnostic tool for malaria diagnosis in Peru^19,34^. This scenario provides a unique opportunity to study the evolution of gene deleted parasites under a scenario of non-significant diagnosis selection pressure.

In this study, a conventional PCR method was used to detect *pfhrp2/3* deletions^5^. The status of *pfhrp2/3* genes were then verified by protein expression levels measured using commercially available HRP2 and pLDH detection ELISA kit^27,28^ and our results from PCR and ELISA were consistent. Genotyping was carried out for a subset of samples in order to understand the evolution of gene deleted parasites in relation to genetic diversity, genetic relatedness and population structure of the parasites. Seven neutral microsatellite markers genotyping was used as the same set of markers and experimental conditions had been used to study *pfhrp2/3* deleted parasites globally including parasites reported in Peru during earlier years^6^ and genotype data are available to us for genetic comparative analysis.

Our results shows that despite the lack of RDTs driven selective pressures, there is a high proportion of dual *pfhrp2/3*-deleted parasites (67.0%) and the prevalence of deletions seem to be heterogeneous across study sites. However, there is a clear trend of increasing prevalence of dual *pfhrp2/3*-deleted parasites with time in the study area. Genotyping data demonstrated that this increase was due to a clonal expansion of a single haplotype (H8) that has deleted both *pfhrp2* and *pfhrp3* genes.

Our results indicate that the spread of this haplotype was rather fast and started with a rapid decrease in the prevalence of non-deleted parasites from 78.6% in 2011 to 26.7% in 2012 and an increase in the prevalence of single *pfhrp2* deleted parasites from 7.1 to 46.7%, . These changes were followed by a rapid increase in the frequency of dual deletions from 22.2% in 2012 to 59% in 2013, although sample numbers in some of earlier years were relatively small.

In this regard, microsatellite data shows that all strains collected since 2011 until 2018 were closely related and did not cluster with lines collected in Peru in the early 2000s. This result matches previous studies that showed a change in circulating parasites in Loreto. Specifically, genotyping data from 1999 and 2000 showed the presence of five circulating *P. falciparum* lineages in Loreto (A, B, C, D and E)^23^. However, genotyping of samples from 2006/2007 showed admixture and appearance of a new hybrid lineage (B/C and C/D)^23^.

Our results, showed that dual *pfhrp2/3-*deleted parasites were closely connected and temporal data supports dominance of the H8 haplotype harbouring dual deletions and its persistence and expansion over time. Possible explanations for this expansion are as follows:

Firstly, decades of effective malaria control programs have brought down the prevalence of *P. falciparum* hence low MOI in the parasite population. This could result in less competition and recombination between strains and lead to a slow turnover of circulating strains.

Secondly, the expansion of H8 could also be explained by selective advantage for this particular haplotype. For instance, H8 parasites could harbour drug resistance mutations against antimalarials commonly used in the region. Unfortunately, our study did not assess the prevalence of these markers on our population. However, previous studies conducted in Peru have not found evidence of resistance against artemisinin nor any partner drug, the main antimalarial used in in Peru^35^.

Diagnostic evasion against RDTs could be another possible explanation for H8 expansion. However, this is highly unlikely as the last reported use of RDTs in Loreto for confirmatory diagnosis was 2006 within the PAMAFRO project^17^ and RDTs showed a low performance with 54% sensitivity for *P. falciparum* detection^34^.

Finally, it is also possible that the change and rapid expansion of the H8 haplotype could be due to lower immune response in the community against a new strain. The success of PAMAFRO may also resulted in a waning of immunity against parasite infections. Our data strongly support this possibility as the circulating lineages, predominantly H8, in our study did not cluster with any of the earlier strains from the same region. Therefore, it is likely that this newly introduced/emerged dual *pfhrp2/3*-deleted strain could have a selective advantage to evade immune responses and rapidly replace the previous circulating strains.

This final hypothesis is further supported by a line of evidence that a change in the circulating *P. falciparum* parasite population occurred in Loreto after 2006 with the report of a new lineage (Bv1) harboring the *pfdhfr* 50R/51I/108N profile^36^. This profile coincides with the Bv1 line which was found first in isolates from Bolivia (1994) and then in Brazil (1997) and Venezuela (1998)^37^. In addition to *pfdhfr,* this line also holds drug resistance associated mutations in *pfdhps, pfcrt* and *pfmdr1*, as well as deletions on *pfhrp2*. Furthermore, this line has been responsible for different outbreaks in Peru^24,38^ and a closely related line has been typed by whole genome sequencing in recent samples in what seems to be a replacement of circulating lineages^39^.

In this regard, our microsatellite data shows that the H8 haplotype is closely related but not completely identical to Bv1. Thus, suggesting that H8 likely emerged after the 2005–2010 PAMAFRO project. It is also likely that a combination of these factors facilitated the rapid clonal expansion of dual *pfhrp2/3* deleted parasites in the study area.

Our study had some limitations that need to be addressed including the relative small sample size for some of the years, the lack of more recent samples after 2018 and the gap on drug resistance markers. In this regard, there is a need for additional studies that identify selection signatures among *pfhrp2/3-*deleted parasites and provide insights into the biological advantage that is linked to the deletion.

In conclusion, we confirmed that there was an increasing trend of dual *pfhrp2/3-*deleted parasites from 2011 until 2018 in Iquitos that is associated with the spread of a new parasite line (H8) that has replaced previously circulating lineages in Peru. The spread of H8 and dual deleted parasites in Iquitos was unlikely driven by RDTs use and seems to be linked to previous malaria eradication efforts conducted In Peru. Our results suggest that the most likely scenario is that the spread of pfhrp2/3 deletions had hitch-hiked on to the clonal expansion of a new strain to which there is little immunity in the community. Our results highlight the need for continuing surveillance in the region to monitor potential changes in circulating parasite populations and their *pfhrp2/3* deletions pattern/trend resulting from changes in diagnostics, case management policies or derived from the COVID19 pandemic.

## Supplementary Information

Supplementary Information.

## Data Availability

The dataset generated during and/or analyzed during the current study is available from the corresponding author on reasonable request.
