# Supplementary material for: Spatiotemporal dynamics of Plasmodium falciparum histidine-rich protein 2 and 3 deletions in Peru
Source: Sci Rep. 2022 Nov 18;12:19845. doi: 10.1038/s41598-022-23881-8 (PMC9674673; doi:10.1038/s41598-022-23881-8)
Supplement: Supplementary file 1 — Supplementary Information. [file 41598_2022_23881_MOESM1_ESM.pdf]

Supplementary files

Spatiotemporal dynamics of *Plasmodium falciparum* histidine-rich protein 2 and 3 deletions in Peru

Hugo O. Valdivia, Karen Anderson, David Smith, Cielo Pasay, Carola J. Salas, Greys Braga, Carmen M. Lucas, Stephen E. Lizewski, Christie A. Joya, Jennifer M Kooken, Juan F. Sanchez, Qin Cheng.

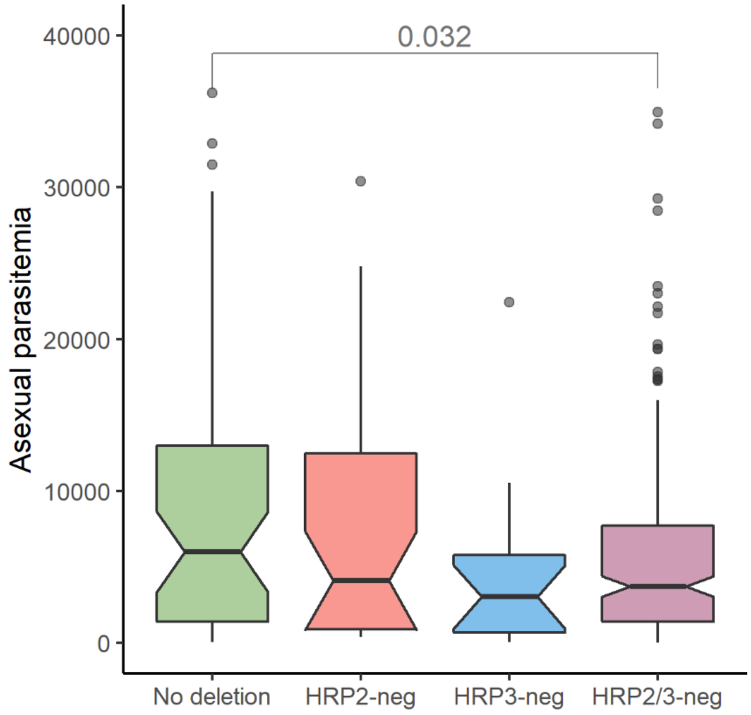

S1 Fig: Peripheral parasitemia determined by microscopy in participants infected with *P. falciparum* with different *pfhrp2/3* gene status. A significant difference was found in parasitemia levels between non-deleted versus dual *pfhrp2/3*-deleted samples according to the pairwise Wilcoxon rank sum test with FDR correction (p=0.032).

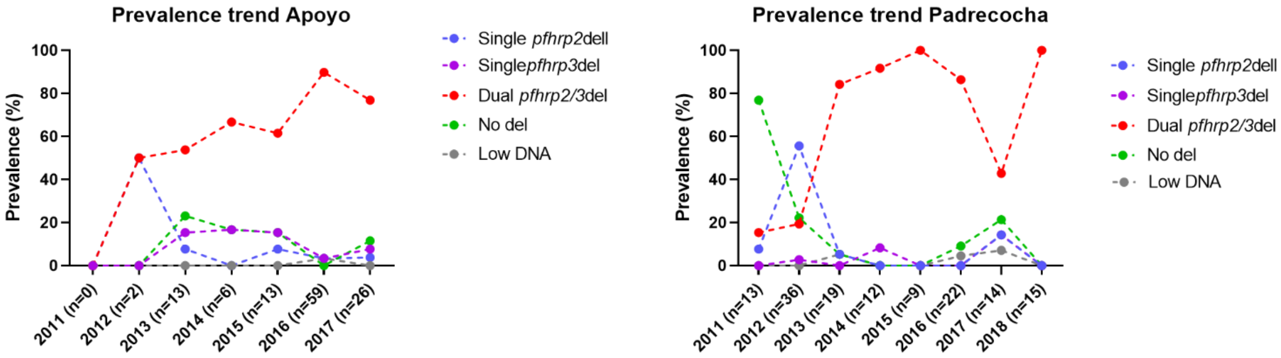

S2 Fig: Prevalence trend of *pfhrp2/3* deletions at Apoyo Hospital (n=119) and Padrecocha (n=140) between 2011 and 2018.

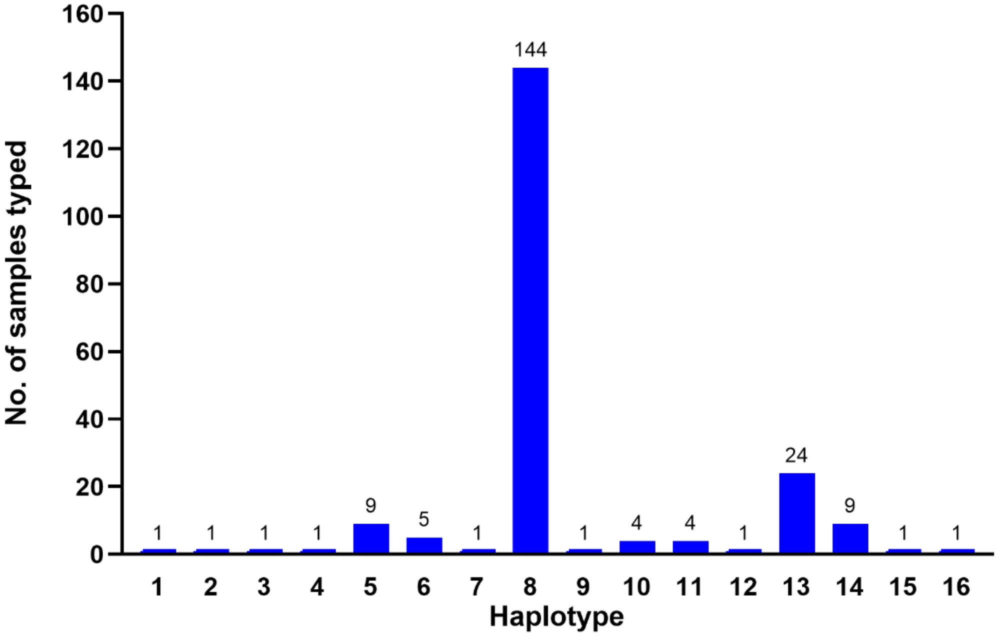

S3 Fig: Haplotype frequency of Peruvian samples. A total of 208 haplotypes (203 single clone +5 polyclonal) were constructed from 203 samples, of which 16 unique haplotypes were obtained.

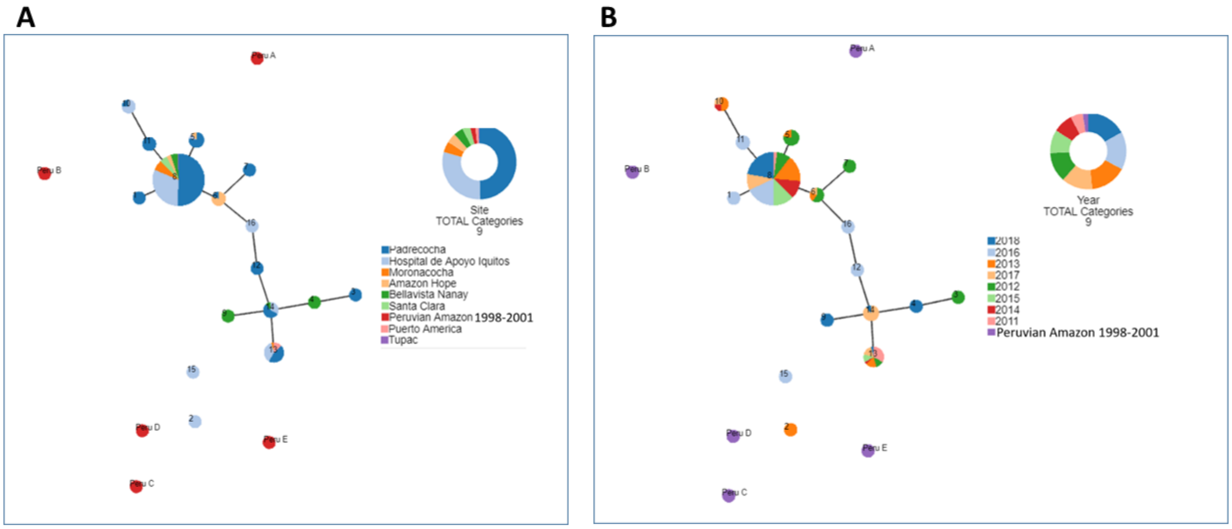

S4 Fig: A) Genetic relatedness of parasites according to study sites. Parasites collected from different study sites clustered together also suggesting a clonal expansion of parasite at these locations. B) Genetic relatedness of parasites according to year of

collection. Parasites collected from different years were clustered together suggesting the persistence of this parasite strain over time and a slow turnover of parasite strains in the population.

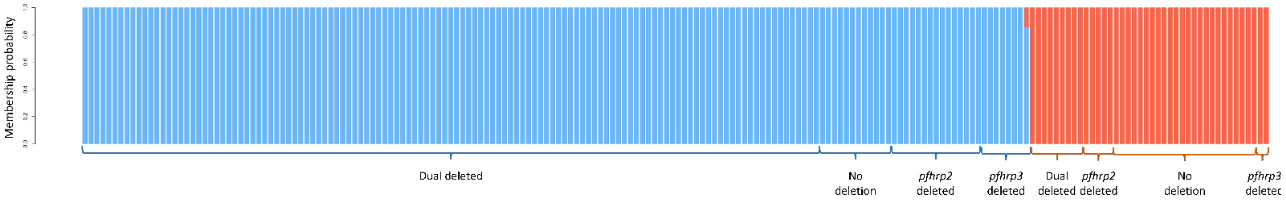

S5 Fig: DAPC with k-means clustering analysis showing the presence of two clusters. Cluster 1 accounted for 90% of deleted parasites whereas cluster 2 accounted for 67.5% of non-deleted parasites.

S1 Table: *Pfhrp2/3* gene deletions by exons. The table shows the proportions of samples having deleted either exon1 or exon2 or both exons among *pfhrp2/3* deleted samples per collection site. Del is short for deletion and Ex, short for exon.

Location	Sample tested	***Pfhrp2***								***pfhrp3***							
		***pfhrp2* del**	**Ex1 del**		**Ex2 del**		**Both Ex del**		**No del**	***pfhrp3* del**	**Ex1 del**		**Ex2 del**		**Both Ex del**		**No del**
		n	n	%	n	%	n	%	n	n	n	%	n	%	n	%	n
**Amazon Hope**	10	2	0	0.0	2	100.0	0	0.0	7	1	0	0.0	0	0.0	1	100.0	9
**Bellavista Nanay**	10	10	0	0.0	0	0.0	10	100.0	0	10	1	10.0	0	0.0	9	90.0	0
**Hospital de Apoyo Iquitos**	117	99	3	3.0	7	7.1	89	89.9	18	102	8	7.8	3	2.9	91	89.2	15
**Moronacocha**	24	18	1	5.6	2	11.1	15	83.3	6	18	1	5.6	1	5.6	16	88.9	6
**Padrecocha**	137	109	1	0.9	29	26.6	79	72.5	28	88	6	6.8	9	10.2	73	83.0	49
**Puerto America**	5	2	0	0.0	0	0.0	2	100.0	3	2	0	0.0	0	0.0	2	100.0	3
**Santa Clara**	12	9	0	0.0	1	11.1	8	88.9	3	10	0	0.0	1	10.0	9	90.0	2
**Tupac**	2	1	0	0.0	0	0.0	1	100.0	1	1	0	0.0	0	0.0	1	100.0	1
**Total**	317	250	5	2.0	41	16.4	204	81.6	66	232	16	6.9	14	6.0	202	87.1	85

S2 Table: Per year prevalence of *pfhrp2* and *pfhrp3* deletions in samples collected between 2011 and 2018.

**Year**	**Sample numbers tested**	Single *pfhrp2* deletion		Single *pfhrp3* deletion		Dual *pfhrp2/3* deletion		No deletion		Low DNA
		n	%	n	%	n	%	n	%	n
**2011**	**14**	1	7.1%	0	0.0%	2	14.3%	11	78.6%	0
**2012**	**45**	21	46.7%	2	4.4%	10	22.2%	12	26.7%	0
**2013**	**39**	4	10.3%	2	5.1%	23	59.0%	9	23.1%	1
**2014**	**18**	0	0.0%	1	5.6%	15	83.3%	2	11.1%	0
**2015**	**22**	1	4.5%	2	9.1%	17	77.3%	2	9.1%	0
**2016**	**81**	2	2.5%	2	2.5%	72	88.9%	2	2.5%	3
**2017**	**42**	3	7.1%	4	9.5%	28	66.7%	6	14.3%	1
**2018**	**63**	1	1.6%	2	3.2%	50	79.4%	8	12.7%	2
**Total**	**324**	33	10.2%	15	4.6%	217	67.0%	52	16.0%	7
